# Informed Written Consent for Orthopaedic Trauma in the Emergency Setting at a Tertiary Referral Centre: A Closed-Loop Audit

**DOI:** 10.7759/cureus.19460

**Published:** 2021-11-11

**Authors:** Martin S Davey, Matthew G Davey, Kunal Mohan, Conor S O'Driscoll, Colin G Murphy

**Affiliations:** 1 Trauma and Orthopaedics, Galway University Hospitals, Galway, IRL

**Keywords:** orthopaedic trauma, patient consent, quality improvement projects, clinical audit system, ­trauma, orthopaedic surgery

## Abstract

Introduction

The purpose of this investigation was to perform an audit of the standards of consent forms in which patients sign prior to operative intervention for orthopaedic trauma in an emergency setting in our institution, with comparison to the *‘Orthopaedic Surgical Consent’ *standards, as set by the *American Association of Orthopaedic Surgeons (AAOS)*. If required, the investigator aimed to close the loop in this audit by educating orthopaedic surgeons on the necessary standards of obtaining written consent for orthopaedic trauma.

Methods

Following being granted approval by our institutional audit committee, a pre-intervention cycle was performed to assess the quality of consent obtained in written format using electronic patient records in consecutive patients over a four-week period. Following the analysis of this data, an education session was provided for all orthopaedic doctors responsible for obtaining informed written consent from patients who are planned to undergo operative management of a soft tissue or bony injury by the trauma and orthopaedic service in the emergency setting. Thereafter, a post-intervention cycle was performed with subsequent descriptive analysis using the GraphPad software.

Results

In the pre-intervention audit cycle, all included (n = 107) consent forms (100%) correctly included the patient’s name, date of birth (DOB) and institutional board number (BN). However, only 79 consent forms (74.5%) were completed without using abbreviations or acronyms of any kind, whilst 81 consent forms (76.4%) were completed without correctly stating the side or site of the planned intervention. In the post-intervention cycle, all included (n = 40) consent forms (100%) correctly included the patient’s name, DOB and institutional BN. Additionally, a total of 37 consent forms (92.5%) were correctly completed without using abbreviations or acronyms of any kind (74.5% versus 92.5%, p = 0.02). Furthermore, a total of 39 consent forms (97.5%) were completed correctly stating the side or site of the planned intervention (76.4% versus 97.5%, p = 0.0015).

Conclusion

This closed-loop audit found that the quality of informed consent obtained by orthopaedic surgeons in the emergency setting might potentially be significantly improved with at least one virtual education session. Such simple education sessions may potentially improve the documentation of the planned potential operative intervention by orthopaedic surgeons for cases of orthopaedic trauma to ensure patient safety is optimised. As the turnover of non-consultant hospital doctors is high in university teaching hospitals, regular education sessions on such topics may introduce a cultural shift in maintaining high standards when marking and consenting patients in the emergency setting.

## Introduction

Referrals to the trauma and orthopaedic service account for a large portion of referrals in Irish emergency departments (ED) annually, with 10% of such referrals representing patients presenting following orthopaedic trauma [1]. As a great variety of injuries occur in many soft tissue and bony injuries, a number of management pathways currently exist in our trauma and orthopaedic services. Of these, most patients tend to be managed non-operatively via outpatient department (OPD) and virtual fracture clinic (VFC) pathways, with a smaller portion being managed operatively by trauma and orthopaedic surgeons in the operating theatre [2,3].

For those who suffered traumatic soft tissue and bony injuries requiring operative intervention, informed consent represents an important aspect of peri-operative care [4]. With respect to this, a number of aspects are required in ensuring informed consent has been adequately acquired from patients or their next of kin or guardian in a smaller portion of patients. Although informed consent in other settings may be obtained in oral or implied formats, operative intervention requires informed consent in written format with a number of key aspects to ensure the validity of the consent in question; these include but are not limited to patient name, date of birth (DOB), hospital identity or board number (BN), operation in question (without abbreviation) and the side or site of their body in which the planned procedure should take place [5]. Despite this as well as previously published Montgomery standards for informed consent [6], previous literature has demonstrated that a significant number of consent forms utilized in orthopaedic operating theatre are incorrectly filled by orthopaedic surgeons [7].

Therefore, it is the belief of the authors of this departmental audit that these basic features are often lacking on the consent forms signed by patients in our tertiary referral centre for orthopaedic trauma [8]. The purpose of this investigation was to perform an audit of the standards of consent forms in which patients sign prior to operative intervention for orthopaedic trauma in an emergency setting in our institution, with comparison to the ‘Orthopaedic Surgical Consent’ standards, as set by the American Association of Orthopaedic Surgeons (AAOS). If required, the investigator aimed to close the loop in this audit by educating orthopaedic surgeons on the necessary standards of obtaining consent in written format for orthopaedic trauma. Our primary hypothesis was that approximately one-third of consent forms audited in our initial cycle would omit at least one core aspect of informed consent, and our secondary hypothesis was that an education session would improve the quality of consent forms obtained when closing the loop of our audit thereafter.

## Materials and methods

Following being granted approval on the 9th of September 2021 to audit by the Galway University Hospitals clinical audit board (Audit Number 205, 2021) under the title ‘Informed Written Consent for Orthopaedic Procedures in the Emergency Setting at a Tertiary Referral Centre - Are We Doing Enough?’, a retrospective review was performed of patients who required operative management of acute orthopaedic trauma in the emergency setting in our institution over a four-week period from 12th of July 2021 to 8th of August 2021. For those requiring an emergency orthopaedic procedure, their electronic medical records were accessed by two independent reviewers (MSD and COD) in order to analyse the standards of their consent forms. Thereafter, verification of data collected was performed by a third reviewer (MGD). As this was an audit of standards with no clinical data being collected or analysed, no informed consent for inclusion in this analysis was ethically required from patients themselves prior to audit commencement.

Outcomes of interest included the following: 1) patient name, 2) patient DOB, 3) patient BN at our institution, 4) name of the proposed procedure in non-abbreviated fashion and 5) correct side or site of the proposed procedure stated clearly. The correct site and side of investigation required the named joint/bone to be spelled, with the following being deemed inadequate: 1) no side/site stated, 2) a letter and circle around it denoting left or right limb and 3) general terms in spinal trauma such as ‘lumbar’ without stating the levels.

After preliminary analysis between the principal investigator (MSD) and the senior investigator (CM), it was decided that a further audit cycle would be required to further audit the standards. The audit committee of our institution proposed that the consent forms of a minimum of a further 30 patients be utilized in order to assess for sufficiently powered quality improvement. Therefore, an education session in relation to desired standards of obtaining consent in format was carried out on the 20th of September with all members of the department of trauma and orthopaedics present. A further audit cycle was performed prospectively over a further period from the 21st of September 2021 to the 4th of October 2021.

Following the second cycle of this audit (i.e., closing the loop), statistical analysis was carried out using the GraphPad software (San Diego, CA, USA). Given that our institution represents a tertiary referral centre for a population of 500,000 people, normal distribution was assumed for all statistical analyses. Fisher’s exact test was performed to analyse the differences between the data obtained in the initial audit cycle and the data obtained during the closed-loop cycle. A p-value of less than 0.05 was deemed to be statistically significant.

## Results

Pre-intervention Cycle

Following our initial audit cycle, a total of 147 (53.7% females) underwent operative management of soft tissue or bony injuries following trauma in our institution during the initial audit period. Of these, the consent forms utilized peri-operatively for 106 patients were available for analysis during this audit. All included consent forms (100%) correctly included the patient’s name, DOB and institutional BN. However, only 79 consent forms (74.5%) were completed without using abbreviations or acronyms of any kind, whilst 81 consent forms (76.4%) were completed without correctly stating the side or site of the planned intervention. Overall, a total of 13 consent forms (12.3%) were completed without using abbreviations or acronyms of any kind and failing to state the side or site of the planned intervention.

Post-intervention Cycle

Following our intervention, a total of 57 (55% females) underwent operative management of soft tissue or bony injuries following trauma in our institution during the post-intervention audit period. Of these, the consent forms utilized peri-operatively for a total of 40 patients were available for analysis during this audit. Similar to pre-intervention, all included consent forms (100%) correctly included the patient’s name, DOB and institutional BN. Additionally, a total of 37 consent forms (92.5%) were completed correctly without using abbreviations or acronyms of any kind, which was an 18% improvement from the pre-intervention cycle (74.5% versus 92.5%, p = 0.02). Furthermore, a total of 39 consent forms (97.5%) were completed correctly stating the side or site of the planned intervention, which was a 21.1% improvement from the pre-intervention cycle (76.4% versus 97.5%, p = 0.0015). This indicated that no consent form (0%) was completed without using abbreviations or else failing to indicate the side or site of the planned intervention. This was 12.3% lower than the pre-intervention cycle (12.3% versus 0%, p = 0.02).

The results of the pre- and post-intervention cycles are presented in Table 1.

**Table 1:** Results Abbrevi.: abbreviation, acron.: acronym

	Cycle 1	Cycle 2	P-value
Total	106	40	-
Patient name	106 (100%)	40 (100%)	>0.99
DOB	107 (100%)	41 (100%)	>0.99
BN	108 (100%)	42 (100%)	>0.99
Abbrev./acron.	79 (74.5%)	37 (92.5%)	0.02
Side/site	81 (76.4%)	39 (97.5%)	0.0015

## Discussion

The most important finding from this closed-loop audit was that the quality of informed consent obtained by orthopaedic surgeons in the emergency setting may be significantly improved through education. Simple education sessions may potentially improve their documentation of the planned potential operative intervention for cases of orthopaedic trauma to ensure patient safety is optimised.

Obtaining informed consent in the peri-operative setting remains a challenging aspect of patient care on an institutional basis, with many factors such as consent form readability, patient reading level and cognition playing pivotal roles in the consent process [9]. Furthermore, Kenyon et al. found high rates of heterogeneity between all 24 hospitals in Ireland at present, with 88% of centres using unique consent forms for obtained informed written consent [10]. Additionally, they noted a lack of uniformity of both format and content amongst the consent forms in each of the elective orthopaedic hospitals in Ireland, before suggesting standardisation of such consent forms amongst all institutions may represent an opportunity to minimise potential risks of litigation for orthopaedic surgeons in the future [10].

Despite the ever-growing body of literature in relation to consent form design and standardisation, the delivery of informed consent in the written format must be clear, concise and without abbreviation [11,12]. Furthermore, Sivanadarajah et al. reported that the standardised consent forms endorsed by the British Orthopaedic Association are significantly more readable and understandable than heterogeneous institutional alternatives [9]. To address this, the authors note the findings of Pomeroy et al. who advocate for procedure-specific consent forms for elective orthopaedic procedures in the elective setting, with significantly higher rates of patient recall of risk factors found at four weeks post-procedure [13]. However, this is often not applicable to orthopaedic trauma, as each case presenting to the ED is independent and unique to the last [14].

Although the layout of consent forms utilised for obtaining informed consent plays a major role in the consent process, one must consider education in relation to the topic. With high turnover in university teaching hospitals with non-consultant hospital doctor staff rotating through many departments, it remains difficult to establish a formal culture of consenting. Therefore, online teaching sessions serve the purpose of attempting to reinforce the Hawthorne effect. Increasing literature reports that virtual patient education results in improved patient recall of the consent process and procedure in question [13,15]. However, no such data is available in relation to orthopaedic surgeons when obtaining informed consent in written form [12,16]. Close observation of more senior colleagues, textbook review of potential operative approaches and complications or even virtual or electronic learning offer some understanding to trainee doctors in obtaining informed consent; however, Halstedian models often still apply [17,18]. Our study found that the use of videoconference style teaching resulted in significant improvements in the quality of information documented during the informed consent process by orthopaedic surgeons in the emergency setting. Therefore, perhaps offering virtual education sessions not only to patients but also to orthopaedic trainees may optimise the quality of written informed consent obtained in the future.

Limitations

Although this closed-loop audit ultimately has improved the standards that it had aimed to, it is not without its limitations. Firstly, only one investigator carried out both the pre- and post-intervention audit cycles, which may introduce potential bias. Secondly, this was completed over a short time period of approximately two months. Thirdly, data was collected in a binary fashion, with numerous errors of the same description being analysed as definitively being correct or incorrect, with no spectrum applied to the data collection. Furthermore, some patients’ consent forms were excluded, as they were not readily available to the investigators. Finally, our education session was confined to a videoconference platform of approximately 90 minutes in duration, with the results of the initial pre-intervention cycle being shared with all participants, a PowerPoint presentation of potential improvement strategies and a question and answer session. 

## Conclusions

This closed-loop audit found that the quality of informed consent obtained by orthopaedic surgeons in the emergency setting might potentially be significantly improved with at least one virtual education session. Such simple education sessions may potentially improve documentation of the planned potential operative intervention by orthopaedic surgeons for cases of orthopaedic trauma to ensure patient safety is optimised. As the turnover of non-consultant hospital doctors is high in university teaching hospitals, regular education sessions on such topics may introduce a cultural shift in maintaining high standards when marking and consenting patients in the emergency setting.
